# Supplementary material for: Co-Infection of L. monocytogenes and Toxoplasma gondii in a Sheep Flock Causing Abortion and Lamb Deaths
Source: Microorganisms. 2022 Aug 15;10(8):1647. doi: 10.3390/microorganisms10081647 (PMC9415574; doi:10.3390/microorganisms10081647)
Supplement: Supplementary file 1 [file microorganisms-10-01647-s001.zip › microorganisms-1772645-SI.pdf]

Supplementary Material

Materials and Methods

Necropsy and histological protocols

They were fixed in 10% neutral buffered formalin and embedded in paraffin. Then the samples were stained with hematoxylin and eosin (HE). For the labelling indirect immunofuorescence (IF) and confocal laser scanning microscopy (CLSM) investigations, 5µm-thick tissue sections of brain presenting microscopic lesions were incubated with specific monoclonal anti-*Listeria monocytogenes* antibody (dilution 1:50) as primary antibody. Finally, section were mounted using antifade medium with 4', 6-diamidino-2-phenylindole (DAPI, Vector Laboratories, Inc.) and imaged using a Leica TCS SP5 II confocal laser microscope.

Microbiological testing

Specimens were stored in sterile containers and then transported and stored before testing at 4 °C, not later than 24 hours from the collection. The tissue or organ samples taken were streaked on three gar media (Blood agar, Mannitol Salt Agar and MacConkey Agar) and all media were incubated in aerobic mecroaerophilic and anerobic conditions at 37 +or- 1C for a maximum of 72 hours. After 24h and 72 h incubation the sold agar media were checked for colony growth (Koneman's, 2019; Krieg, et al., 2005; Quinn et al., 2013; Wadsworth, 2010).

Molecular detection

DNA extraction

For the organ tissue approximately 2 cm^3 of the samples were placed into stomacher bags with the addition of 10 mL of PBS pH 7.4. They were then homogenated and centrifuged at 2000 g for 10 min. 300 µL of supernatant was used for extraction.

PCR per real time abortigen agent detection

For tissue PCR the reaction mix was prepared using the Genesig® Advanced Kit (Genesig, York House, School Lane, Chandler's Ford, UK) in a final volume of 20 µl consisting of 5 µl of extracted DNA and 15 µl of master mix, according to the manufacturer's instructions. Enzyme activation step at 95°C for 2 min, 50 cycles of denaturation at 95°C for 10 sec and data collection at 60°C for 60 sec. A positive control (K+) and a No-Template Control (NTC) were included in each run.

Serological testing

Blood samples were taken for serological testing with vacutainer system without anticoagulant from the carotid artery, allowing blood to clot for serum separation. Blood was refrigerated on ice for 2 h, centrifuged at 2400 x g for 10 min at 4° C and the serum was stored at -20° C until further analysis.

Immunological detection of *L. monocytogenes*

The "Listeria sheep Test" ELISA kit (DIATHEVA, Italy) was used for *L. monocytogenes* analysis of the sera. Briefly, 100µl of the 1: 100 diluted samples sera were dispensed into the wells and incubated at 37°C for 1 hour, in the dark. After washings, 100µl/well of secondary antibody-horseradish Peroxidase (HRP) was dispensed and incubated at 37°C for 1 hour, in the dark. The antigen-antibody reaction was detected by adding 100µl per well of ABTS solution at room temperature for 20 minutes, protected from light. Finally, optical densities (OD) were acquired with a microplate reader at 450nm. All samples were tested in duplicate.

Results were expressed as S/P calculated using the following formula:

$$\frac{S}{P} = \frac{(sampleA(O.D.mean) - negativecontrol(O.D.mean))}{(positivecontrol(O.D.mean) - negativecontrol(O.D.mean))}$$

The presence or absence of antibody against *L. monocytogenes* was determined using the following cut off depending on the purpose:

1) Prevalence of infection and demonstration of freedom from infection in individual animals: sera with S/P < 0,420 are Negative, sera with S/P > 0,420 are Positive;
2) Confirmatory diagnosis of suspect of clinical cases: sera with S/P < 0,450 are Negative, sera with S/P > 0,450 are Positive;
3) Determination of immune status in individuals or population post vaccination: sera with S/P < 0,380 are Negative, sera with S/P > 0,380 are Positive.

For the purpose of this study the sera values sera with S/P < 0,450 are Negative, sera with S/P > 0,450 are Positive;

Immunological detection of *Chlamydia psittaci* var. *ovis*

Sera were tested for detection of antibodies versus *Chlamydia psitatci* var. *ovis* by Complement Fixation Test.

Sera from samples was taken and diluted 1:16 in Veronal buffered saline (IDvet, France), then it was inactivated in a water bath at 60°C ± 2°C for 30 minutes.

Inactivated sera were at dilutions ratio 1/2 between 1:16 dilution to 1:64 in VBS. Standard 96-weel microtiter plates with round (U) bottoms were used. Each well was filled with 25±l microliters of sera, 25±l microliters of antigen (IZSAM, Italy), diluted to working strength, and 25±l microliters of complement (IDvet, France) diluted to the number unit required.

At the same time controls performed with reference sera (IZSAM, Italy), added to the plate. Control wells were prepared with: I) diluent + sera + complement (anti-complementary control); II) diluent + antigen + complement; III) diluent + complement; IIII) diluent only.

Plates were incubated at 37°C ± 2°C for 30 minutes, thereafter 25±l microliters of sheep red blood cells (SBRCs, IZSAM, Italy), sensitized with an equal volume of rabbit anti-SBRC serum, was added in each well and centrifuged at 1500rpm for 4 minutes at 5°C ± 3 °C. The degree of haemolysis was compared with reference sera and controls.

Sera showing 100% fixation were considered as positive and the reactivity of the test sample was expressed by the titer, i.e. the highest dilution of the serum that provides 100% fixation (++++). Sera showing less than 100% fixation at the first dilution (1:16) were considered negative.

Immunological detection of *C. burnetii*

Sera were tested with indirect ELISA for detection of antibodies versus *C. burnetii* (ID Screen Q Fever Indirect Multi-species, IDvet, France).
100µl of sample was diluted to a final dilution of 1:50 with a dilution buffer and incubated 45 minutes at room temperature in the ELISA microplate. After washing, 100µl/well of a secondary-HRP (1:10 dilution) was added and incubated for 30 minutes at room temperature, protected from light. For the detection of the antigen-antibody complex the TMB substrate was added and the reaction was stopped with STOP solution. The colorimetric reaction (expressed as optical density, O. D.) was read with a microplate reader at a wavelength of 450nm.
For each sample the percentage S/P was calculated using the following formula:

$$\frac{S}{P}\% = \frac{O.D.sample - O.D.negativecontrol}{O.D.positivecontrol - O.D.negativecontrol} x100$$

Samples with S/P% less than or equal to 40% were considered Positive, greater than 40% and less than 50% were considered Doubtfull, greater than 50% and less than 80% were considered Positive and samples with S/P% greater than 80% were considered Strong Positive.

Immunological detection of *Neospora caninum*

Sera were tested by competitive ELISA kit for detection of antibodies against *N. caninum* (ID Screen *N. caninum* Competition IDvet, France).
100µl of sample was diluted with a dilution buffer to a final dilution of 1:2 and incubated 45 minutes at 37°C ± 2°C in the ELISA microplate. After washing, 100µl/well of a secondary-HRP (1:10 dilution) was added and incubated for 30 minutes at room temperature, protected from light. For the detection of the antigen-antibody complex the TMB substrate was added and the reaction was stopped with STOP solution. The colorimetric reaction (expressed as optical density, O. D.) was read with a microplate reader at a wavelength of 450nm.
For each sample the percentage S/N was calculated using the following formula:

$$\frac{S}{N}\% = \frac{O.D.sample}{O.D.negativecontrol}x100$$

Samples with S/N% less than or equal to 50% were considered Positive, greater than 50% and less than 60% were considered Doubtfull, greater than 60% were considered Negative.

Immunological detection of *Salmonella* abortus ovis

Sera were tested for detection of antibodies for *S.* Abortusovis by serum agglutination test.
Sera were diluted 1:20 in phenol saline solution (PSS) and serial doubling dilutions were performed in polystyrene tubes. Then, the antigen (IZSAM, Italy), appropriately diluted in PSS, was added to each sample. After shaking, the sera were incubated overnight at 37°C ± 2°C.
Positive and negative reference sera (IZSAM, Italy) and reaction controls were prepared using PSS with the addition of 1% negative sheep serum and diluted antigen. Five reaction controls were used to simulate the different degrees of turbidity of the final solution.
A sample was considered serologically negative if it did not show agglutination and a uniform suspension was in the tube, or if at the first dilution the agglutination was lower than 100%. The sample was considered positive if agglutination was present at the bottom of the tube and the supernatant was clear.

Suppl. Results

Serological

Table S1. Results of serological testing

Animal ID	Notes	Chlamydiaceae		*C. burnetii*		*Listeria monocytogenes*		*N. caninum*		*Toxoplasma gondii*		*Salmonella* Abortusovis	
		Feb	July	Feb	July	Feb	July	Feb	July	Feb	July	Feb	July
1	Delivery: One small lamb	1:16	Neg	Neg	Neg	Pos	Neg	Neg	Ø	Pos	Pos	Neg	Neg

2	Delivery: Twins one alive and one dead after three days	1:16	Slaughtered	Neg	Slaughtered	Pos	Slaughtered	Neg	Slaughtered	Pos	Slaughtered	Neg	Slaughtered
3	Delivery: one small lamb	1:16	Slaughtered	Neg	Slaughtered	Pos	Slaughtered	Neg	Slaughtered	Pos	Slaughtered	Neg	Slaughtered
4	Abortion	1:16	Neg	Neg	Neg	Pos	Neg	Neg	Ø	Pos	Pos	Neg	Neg
5	Lamb with neurologic disorders	Ø	Neg	Ø	Neg	Ø	Neg	Ø	Ø	Ø	Neg	Ø	Neg
6	Delivery: One small lamb	Ø	Neg	Ø	Neg	Ø	Neg	Ø	Ø	Ø	Pos	Ø	Neg
7	Control group	Ø	Neg	Ø	Neg	Ø	Pos	Ø	Ø	Ø	Pos	Ø	1:80
8	Control group	Ø	Neg	Ø	Neg	Ø	Neg	Ø	Ø	Ø	Neg	Ø	Neg
9	Control group	Ø	Neg	Ø	Neg	Ø	Neg	Ø	Ø	Ø	Pos	Ø	Neg
10	Control group	Ø	Neg	Ø	Neg	Ø	Neg	Ø	Ø	Ø	Pos	Ø	Neg
11	Control group	Ø	Neg	Ø	Neg	Ø	Neg	Ø	Ø	Ø	Pos	Ø	Neg
12	Control group	Ø	Neg	Ø	Neg	Ø	Neg	Ø	Ø	Ø	Pos	Ø	Neg
13	Control group	Ø	Neg	Ø	Neg	Ø	Neg	Ø	Ø	Ø	Pos	Ø	Neg
14	Control group	Ø	Neg	Ø	Neg	Ø	Neg	Ø	Ø	Ø	Pos	Ø	Neg
15	Control group	Ø	Neg	Ø	Pos	Ø	Neg	Ø	Ø	Ø	Pos	Ø	Neg
16	Control group	Ø	Neg	Ø	Pos	Ø	Pos	Ø	Ø	Ø	Pos	Ø	Neg
17	Control group	Ø	Neg	Ø	Neg	Ø	Pos	Ø	Ø	Ø	Pos	Ø	Neg
18	Control group	Ø	Neg	Ø	Neg	Ø	Neg	Ø	Ø	Ø	Pos	Ø	Neg
19	Control group	Ø	Neg	Ø	Neg	Ø	Neg	Ø	Ø	Ø	Pos	Ø	Neg
20	Control group	Ø	Neg	Ø	Pos	Ø	Neg	Ø	Ø	Ø	Pos	Ø	Neg
21	Control group	Ø	Neg	Ø	Neg	Ø	Neg	Ø	Ø	Ø	Pos	Ø	Neg
22	Control group	Ø	Neg	Ø	Neg	Ø	Neg	Ø	Ø	Ø	Pos	Ø	Neg

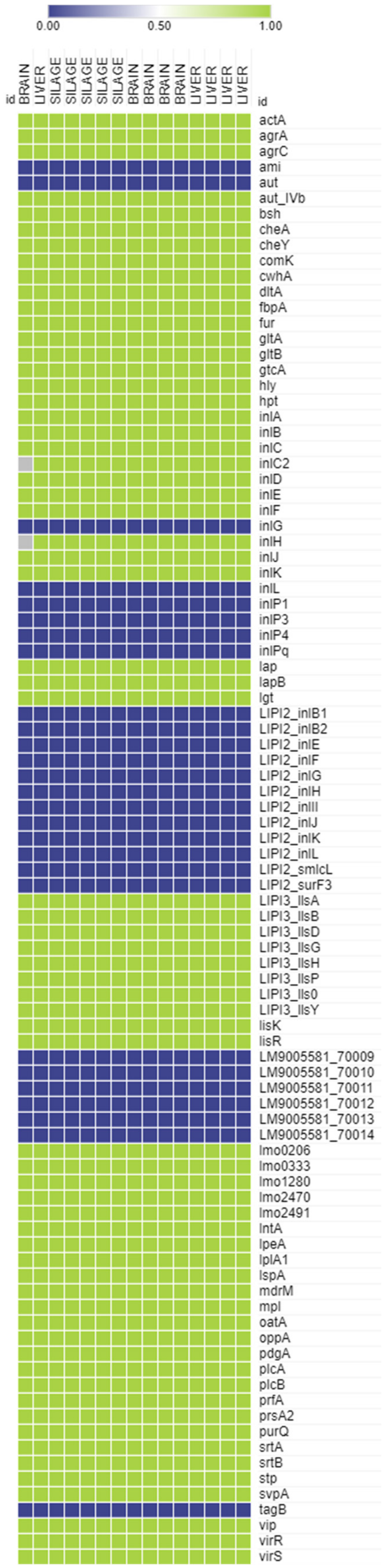

Figure S1
In green virulence gene present, in blue virulence gene missing. In grey undetermined virulence gene in one singleton.

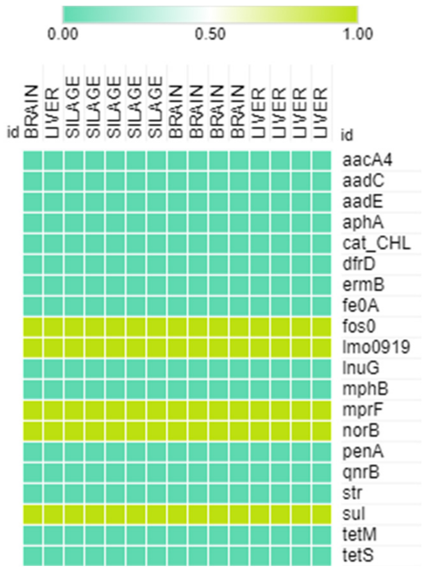

Figure S2

Antibiotic resistance genes. In yellow antibiotic resistance gene present, in green antibiotic resistance gene missing.
